# CMIP6 models informed summer human thermal discomfort conditions in Indian regional hotspot

**DOI:** 10.1038/s41598-023-38602-y

**Published:** 2023-08-02

**Authors:** Krishna Kumar Shukla, Raju Attada

**Affiliations:** Department of Earth and Environmental Sciences, Indian Institute of Science Education and Research Mohali, SAS Nagar, Manauli, Sector 81, Knowledge city, 140306 Punjab India

**Keywords:** Climate sciences, Environmental sciences, Natural hazards

## Abstract

The frequency and intensity of extreme thermal stress conditions during summer are expected to increase due to climate change. This study examines sixteen models from the Coupled Model Intercomparison Project Phase 6 (CMIP6) that have been bias-adjusted using the quantile delta mapping method. These models provide Universal Thermal Climate Index (UTCI) for summer seasons between 1979 and 2010, which are regridded to a similar spatial grid as ERA5-HEAT (available at 0.25° × 0.25° spatial resolution) using bilinear interpolation. The evaluation compares the summertime climatology and trends of the CMIP6 multi-model ensemble (MME) mean UTCI with ERA5 data, focusing on a regional hotspot in northwest India (NWI). The Pattern Correlation Coefficient (between CMIP6 models and ERA5) values exceeding 0.9 were employed to derive the MME mean of UTCI, which was subsequently used to analyze the climatology and trends of UTCI in the CMIP6 models.The spatial climatological mean of CMIP6 MME UTCI demonstrates significant thermal stress over the NWI region, similar to ERA5. Both ERA5 and CMIP6 MME UTCI show a rising trend in thermal stress conditions over NWI. The temporal variation analysis reveals that NWI experiences higher thermal stress during the summer compared to the rest of India. The number of thermal stress days is also increasing in NWI and major Indian cities according to ERA5 and CMIP6 MME. Future climate projections under different scenarios (SSP1-2.6, SSP2-4.5, and SSP5-8.5) indicate an increasing trend in thermal discomfort conditions throughout the twenty-first century. The projected rates of increase are approximately 0.09 °C per decade, 0.26 °C per decade, and 0.56 °C per decade, respectively. Assessing the near (2022–2059) and far (2060–2100) future, all three scenarios suggest a rise in intense heat stress days (UTCI > 38 °C) in NWI. Notably, the CMIP6 models predict that NWI could reach deadly levels of heat stress under the high-emission (SSP5-8.5) scenario. The findings underscore the urgency of addressing climate change and its potential impacts on human well-being and socio-economic sectors.

## Introduction

The rising occurrence frequency of extreme temperature events due to the unprecedented scale of climate change over the past few decades has led to an increase in thermal stress worldwide^1–4^. Under different climatic scenarios, the average global temperature is projected to increase by more than 4 °C after the pre-industrial era in the future^5,6^. The increased thermal stress has a pronounced negative impact on human health, mainly in vulnerable populations such as senior citizens, chronically ill, poorer communities, agriculture, ecosystems, and the economy^7,8^.

The Indian subcontinent experiences frequent heat waves during the summer^9–11^. However, annual surface temperature of the Indian subcontinent shows an increasing trend (0.64 °C per 100 years) during 1901–2016^12^ which will impose significant thermal stress over the region. The rising surface temperatures further favor the increased evapotranspiration (evaporation) from vegetation and water bodies which further increases the specific humidity in the region and its positive feedback for extremely humid heat.) The extreme thermal stress condition majorly depends on high humid conditions than anomalous temperature^13,14^. The increased frequency of human thermal discomfort due to heat extremes has attracted the attention of the scientific community in recent years.

Various thermal stress indices have been used to assess thermal stress conditions worldwide^15–24^. The Universal Thermal Climate Index (UTCI) is a valuable thermal index that considers heat transfer between the human body and the environment, making it suitable for assessing thermal stress across a wide range of heat exchange conditions^25,26^. Studies in Europe have shown an increase of approximately 1 °C in UTCI over recent decades^18,19,22^. Thermal stress assessments based on UTCI in eastern India reveal higher stress levels in areas with manmade infrastructure compared to natural land covers during summer^27,28^. Seasonal climatological data from 1981 to 2019 indicate moderate to strong thermal stress over NWI compared to other regions in India, with a significant rising trend in UTCI^29^. Increased thermal stress associated with climate extremes is a primary cause of mortality in various regions globally^7,16,19,23,30–39^. Therefore, understanding and projecting future thermal stress conditions in India using climate models is crucial.

Climate models, such as those from the Coupled Model Intercomparison Project Phase 5/6 (CMIP5/6), provide valuable tools for assessing future thermal stress scenarios. CMIP5 simulations indicate a consistent increase in heat waves and thermal stress across the Indian Peninsula^40^, with projections of rising frequency and duration of heat waves over central India and NWI^41^. Further, CMIP5 models predict that temperature and heat stress extremes will co-occur in mid-latitudes, surpassing health thresholds in tropical regions^42^. It is, therefore, work performance in India is expected to decline by the end of the century due to elevated summer thermal stress based on CMIP5 projections^43^. Thermal stress indices derived from CMIP6 simulations also indicate significant global increasing trends^44^. To comprehensively assess the current and future thermal discomfort conditions, combining climate change, physiology, and epidemiology is crucial for obtaining robust projections of heat-related health risks^45^.

Previous studies have analyzed thermal stress based on UTCI using the ERA5-HEAT database specifically in NWI^29,39,46^. However, these studies have focused on ‘present climate’ thermal stress conditions in India using reanalysis and in-situ observations. This study is the first attempt to utilize bias-corrected CMIP6 model UTCI data to evaluate historical simulations and understand projected future thermal stress conditions in India, specifically NWI. The findings will contribute to designing mitigation and adaptation strategies to mitigate the negative impacts of thermal stress on society.

The recently released sixth phase of climate models (CMIP6) runs at a relatively higher spatial resolution, offering enhanced insights into both current and future thermal discomfort conditions. A detailed overview of these models is available in Eyring et al.^47^. In the present study, we aim to explore the climatology of thermal stress conditions, trends and their future projections using CMIP6 models over a regional hotspot of India during the summer season. Additionally, we show the future evolution (near and far future) of thermal discomfort conditions under three Shared Socioeconomic Pathways (SSPs): SSP1-2.6, SSP2-4.5, and SSP5-8.5. The manuscript is organized as follows: "Data and methodology" Section discusses the data and methods employed, while "Results and discussion" Section presents the key findings. Finally, "Conclusions" Section comprises the discussions and summary.

## Data and methodology

### Study area

The NWI is, known as the country's breadbasket, a hotspot for thermal discomfort due to several factors. The region experiences frequent heat wave events with increasing trends in frequency, intensity, and duration^9^. These heat waves are associated with persistent high anti-cyclonic flow, clear skies, and depleted soil moisture, intensifying the thermal stress. The region also suffers from a significant aerosol burden^29^, including dust storms and post-harvest burning, which trap heat in the atmosphere and contribute to higher temperatures. With its crucial role in agriculture and the adverse effects of heat waves on crop yield and productivity, NWI’s economy is impacted. These extreme thermal conditions have implications for human health, energy consumption, work performance, and tourism. Considering the severity of thermal stress and its wide-ranging impacts, the region has been selected as a hotspot for evaluating the CMIP6.

### Datasets

The ERA5-HEAT (Human thermal comforT) provides the thermal index (i.e., UTCI) data which is produced by the European Centre for Medium-Range Weather Forecasts (ECMWF) within the Copernicus Climate Change Service. The ERA5-HEAT UTCI is available globally at a high spatio-temporal resolution from 1979 to the present and can be downloaded at the Climate Data Store^48^. UTCI is estimated by considering the heat transfer between the human body and the environment^25^. Therefore, UTCI is extensively used as human biometeorology parameters to assess the relationships between outdoor environment conditions and human well-being^48^. The present study uses the hourly UTCI (°C) data at a spatial resolution of 0.25° × 0.25° from 1979 to 2010.

For this analysis, we utilized data from sixteen bias-corrected CMIP6 models, which are part of the World Climate Research Programme^47^. The UTCI (°C) datasets derived from various CMIP6 models were downloaded^49^ specifically for the summer/hot season (April-May-June-July; AMJJ) from the following source: https://cds.climate.copernicus.eu/cdsapp#!/dataset/sis-extreme-indices-cmip6?tab=overview. During the summer period from 1979 to 2010, daily mean bias-adjusted UTCI data was collected from a single ensemble member (r1i1p1f1) of each of the sixteen CMIP6 models, with varying spatial resolutions (as indicated in Table 1^50–65^)^44,66^. Furthermore, we also obtained projected UTCI data (2011–2100) for the summer season under three different climate scenarios (SSP1-2.6, SSP2-4.5, and SSP5-8.5) from all CMIP6 models to assess future thermal stress projections. The evaluation of CMIP6 UTCI was performed by comparing it with data from ERA5.

**Table 1:** List of 16 CMIP6 coupled earth system models with horizontal resolution.

S. No	CMIP6 model	Horizontal resolution	References
1	ACCESS-CM2 (Australia)	1.875° × 1.25°	Dix et al.^53^
2	ACCESS-ESM1-5 (Australia)	1.875° × 1.24°	Ziehn et al.^54^
3	CanESM5 (Canada)	2.8125° × 2.8125°	Swart et al.^55^
4	EC-Earth3 (Europe)	0.703125° × 0.703125°	Döscher et al.^56^
5	EC-Earth3-Veg (Europe)	0.703125° × 0.703125°	Wyser et al.^57^
6	FGOALS-g3 (China)	2.0° × 2.25°	Li et al.^58^
7	GFDL-CM4 (USA)	1.25° × 1.0°	Held et al.^59^
8	GFDL-ESM4 (USA)	1.25° × 1.0°	Horowitz et al.^60^
9	INM-CM4-8 (Russia)	2.0° × 1.5°	Volodin et al.^61^
10	INM-CM5-0 (Russia)	2.0° × 1.5°	Volodin et al.^62^
11	KIOST-ESM (South Korea)	1.875° × 1.875°	Kim et al.^63^
12	MPI-ESM1-2-HR (Germany)	0.9375° × 0.9375°	Jungclaus et al.^64^
13	MPI-ESM1-2-LR (Germany)	1.875° × 1.875°	Wieners et al.^65^
14	MRI-ESM2-0 (Japan)	1.125° × 1.125°	Yukimoto et al.^66^
15	NorESM2-LM (Norway)	2.5° × 1.875°	Seland et al.^67^
16	NorESM2-MM (Norway)	1.25° × 0.9375°	Bentsen et al.^68^

### Methodology

The seasonal averages were obtained by averaging the daily UTCI from all sixteen CMIP6 models for each summer/hot (April through July) season during 1979–2010. ERA5 derived UTCI available at 0.25° × 0.25° spatial resolution data is considered as a reference dataset to evaluate CMIP6 models. We have then regridded all sixteen CMIP6 models onto the similar spatial grid resolution of ERA5 (0.25° × 0.25°) using the bilinear interpolation technique^67^ for fair comparison and the statistical evaluation of both datasets. The Pattern Correlation Coefficient (PCC) was computed individual CMIP6 model datasets (including the Multi-Model Ensemble (MME) mean of CMIP6 models) and ERA5 at a 95% significance level. The PCC values greater than 0.9 were used to compute the MME mean of UTCI, which was further utilized to determine the climatology and trends of UTCI in the CMIP6 models. Furthermore, we assessed the trends in UTCI for both the CMIP6 MME and ERA5 datasets during the summertime period from 1979 to 2010. To evaluate the presence of a monotonic upward or downward trend in the UTCI over time, we employed the Mann-Kendall (MK) test, which is a non-parametric (distribution-free) test^68,69^. This is a widely used technique to test climate trends^9,70,71^. The statistical significance of the UTCI trends at each grid point for the CMIP6 MME and ERA5 datasets was determined using a two-tailed Student's t-test.

Additionally, we have also computed the strong and very strong thermal stress days over NWI using a threshold of 32 °C (strong heat stress) and 38 °C (very strong heat stress) at daily summertime UTCI during the historical period (1979–2010) as defined by Di Napoli et al.^48^. Furthermore, the very strong thermal stress days were also computed during the near future (2022–2059) and far future (2060–2100) under all three projected climate scenarios (SSP1-2.6, SSP2-4.5, and SSP5-8.5) over NWI. Here, it is important to mention that, recently, various researchers started climate forecasting using AI/ML models. For example, Haq^72^ used the Climate Deep Long Short-Term Memory (CDLSTM) model was developed and optimized to forecast all Himalayan states’ temperature and rainfall values. Facebook’s Prophet (FB-Prophet) model was also implemented to forecast and assess the performance of the developed CDLSTM model. Nevertheless, it is important to note that these aforementioned models primarily rely on data-driven approaches. In contrast, the objective of the present study is to assess dynamical (physical-based) models for forecasting the future progression of thermal stress conditions.

## Results and discussion

### Evaluation of thermal stress climatology

**Figure 1:**
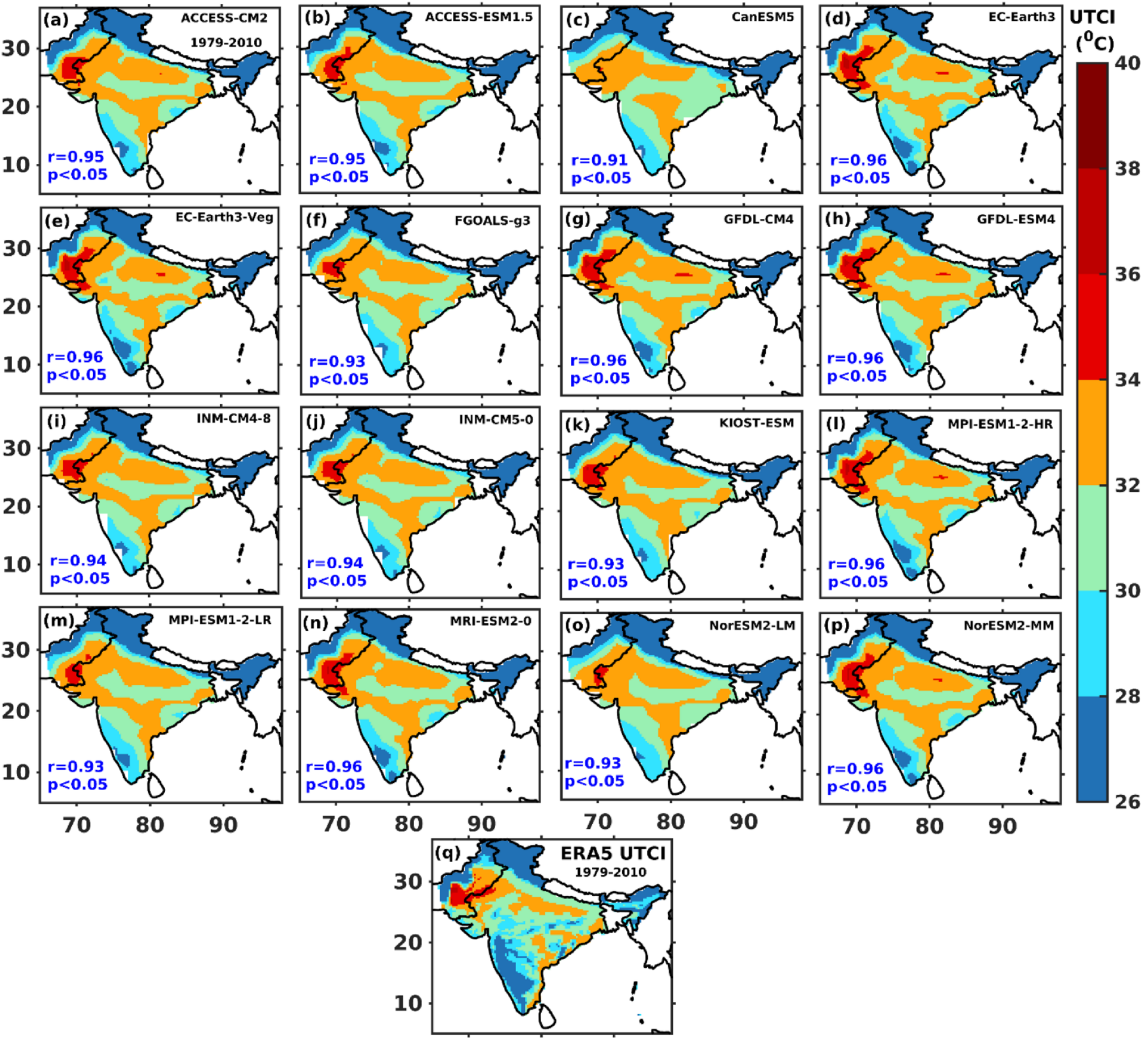
Spatial pattern of climatological UTCI mean of (**a**–**p**) different CMIP6 models and (**q**) ERA5 during summer (AMJJ, 1979–2010) over India. Pattern correlation coefficient (PCC) between individual CMIP6 models and ERA5 is given in (**a**–**p**) at 95% confidence level. The map was generated using MATLAB R2017b (www.mathworks.com). The colormap of figures are taken in MATLAB from (https://www.ncl.ucar.edu/Document/Graphics/color_table_gallery.shtml).

The spatial pattern of climatological (1979–2010) mean UTCI calculated from sixteen CMIP6 models and ERA5 is presented in Fig. 1a–q over the Indian subcontinent during summer. The PCC between ERA5 and sixteen individual CMIP6 models UTCI is given in Fig. 1a–p at 95% confidence level. The CMIP6 models capture the spatial variability of summertime UTCI like ERA5 over India, albeit with some differences. The PCCs between sixteen individual CMIP6 models and ERA5 are statistically significant and greater than 0.9 (Fig. 1a–p). Hence, the MME mean is computed by averaging sixteen individual CMIP6 models. The MME is mainly used due to its better performance than individual models^73,74^.

**Figure 2:**
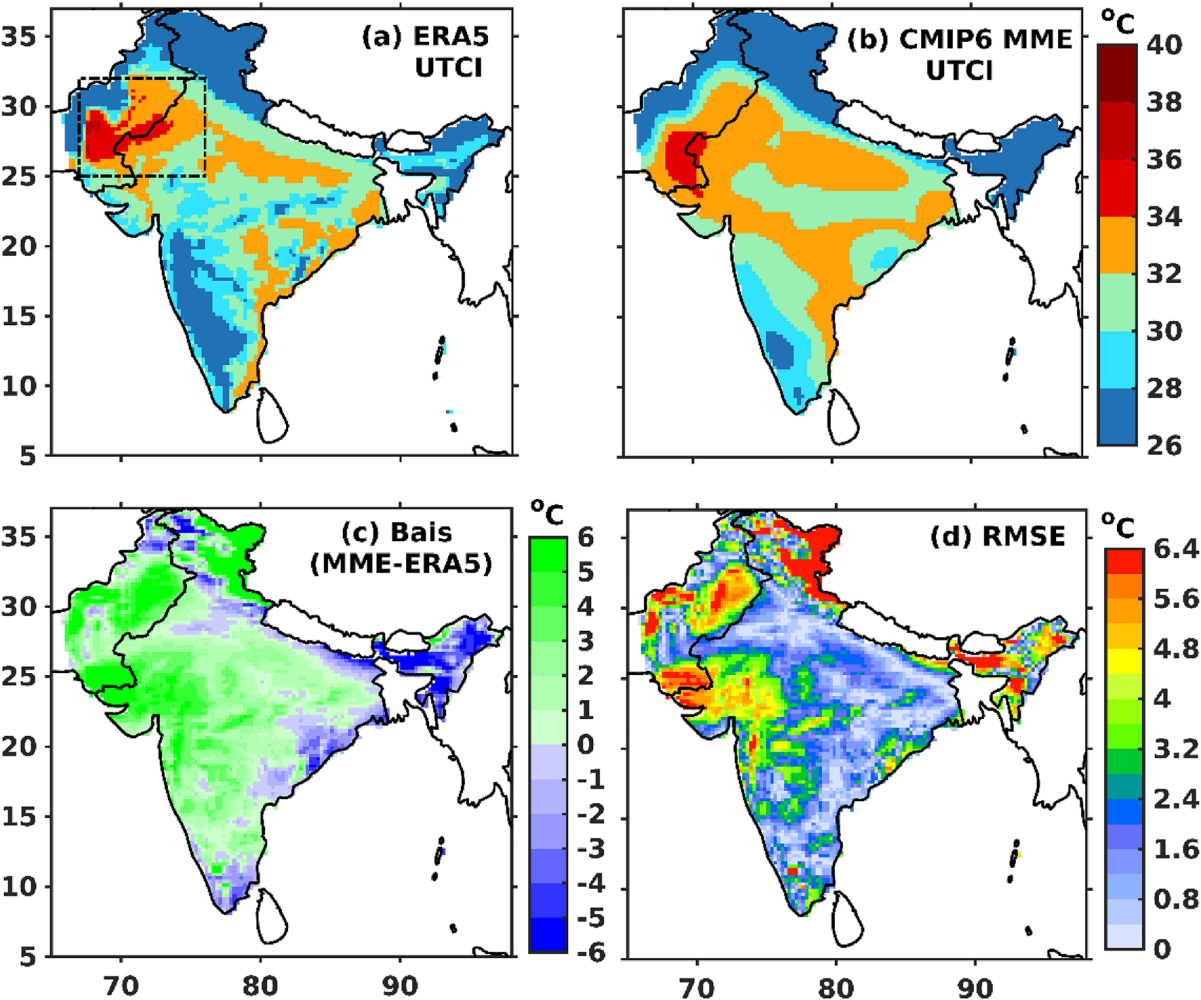
Spatial pattern of summer climatological (1979–2010) mean of UTCI (**a**) ERA5 and (**b**) CMIP6 MME, (**c**) bias between ERA5 and CMIP6 MME, (**d**) root mean square error (RMSE). The dotted black line outlines northwest India (NWI) (25–32°N; 67–76°E) in (**a**). PCC between ERA5 and CMIP6 MME is 0.95 at 95% significance. The map was generated using MATLAB R2017b (www.mathworks.com). The colormap of figures are taken in MATLAB from (https://www.ncl.ucar.edu/Document/Graphics/color_table_gallery.shtml).

The spatial pattern of climatological mean ERA5 and CMIP6 MME UTCI are shown in Fig. 2a,b, and the significant PCC is 0.95 between ERA5 and CMIP6 MME at a 95% confidence level. Figure 2a demonstrates that UTCI is usually greater than 32 °C over NWI compared to other Indian regions. The CMIP6 MME captures the spatial distribution of UTCI (greater than 32 °C) over NWI (Fig. 2b). The summertime ERA5 and CMIP6 MME UTCI demonstrate moderate and strong thermal stress conditions over NWI (hotspot region). The spatial pattern of bias between CMIP6 MME and ERA5 shows that CMIP6 MME usually overestimates the UTCI over NWI compared to ERA5 (Fig. 2c). The root mean square error (RMSE) varies between 0.8 to 6 °C over the entire study region, possibly due to the high overestimation of UTCI in the model (Fig. 2d).

### Spatial and temporal trends

**Figure 3:**
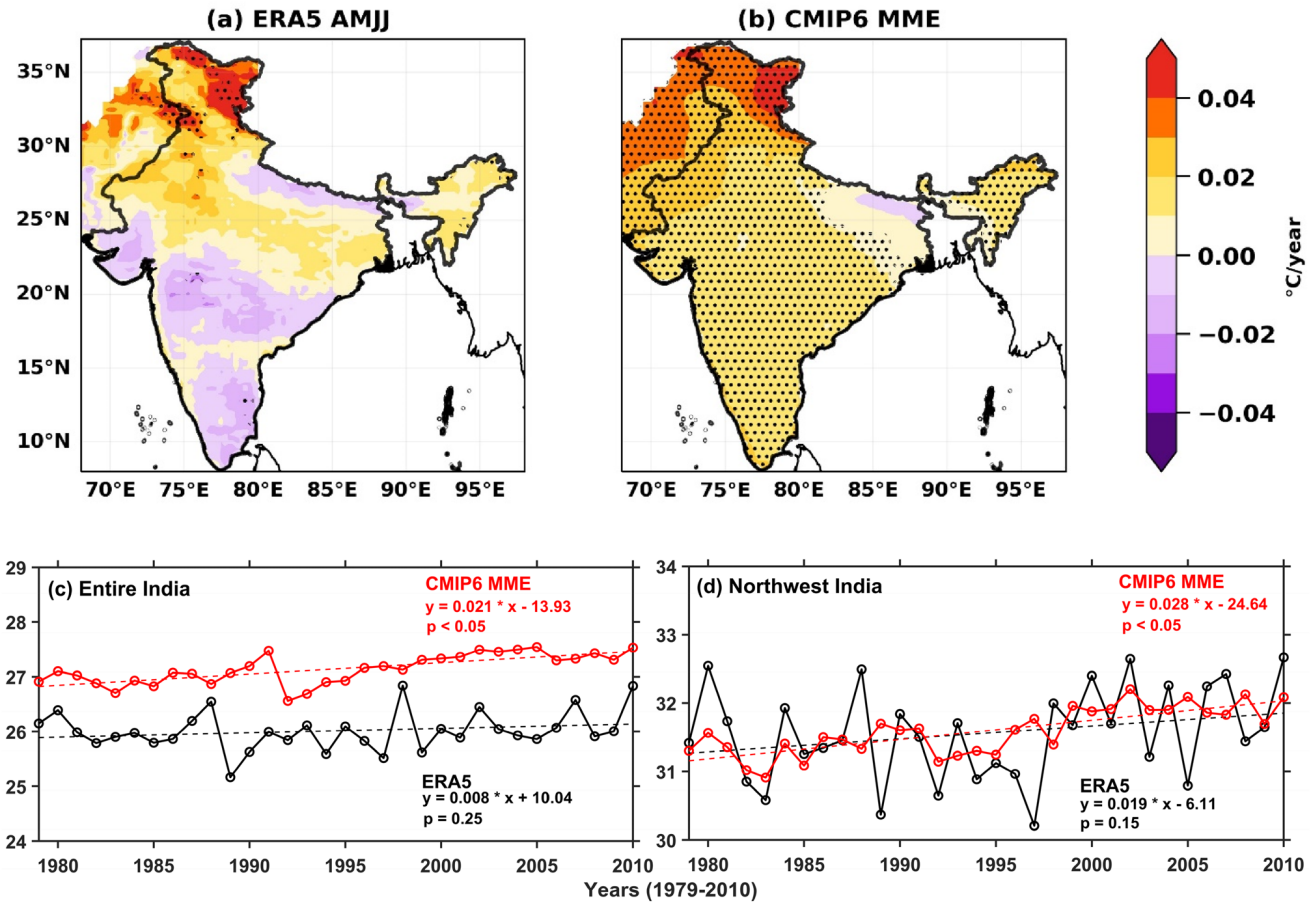
(**a**, **b**) Spatial pattern of trends (°C year^− 1^) in (a) ERA5, (**b**) CMIP6 MME UTCI during the summer season. Seasonal temporal trends of area-averaged ERA5 and CMIP6 MME UTCI for (**c**) the entire Indian subcontinent and (**d**) NWI during the summer season. The spatial (black dots) and temporal trends are statistical significance at 95% confidence level. The map was generated using Python 3.8 (https://www.python.org/downloads/release/python-380/).

The spatial distribution of UTCI trends (ERA5 and CMIP6 MME) are shown in Fig. 3a, b during summer (1979–2010). The ERA5 and CMIP6 MME UTCI shows rising trends over NWI, which indicates that CMIP6 MME can capture the UTCI trend like ERA5. The UTCI trends from both are generally greater than 0.2 °C per decade. ERA5 UTCI trends are significant at a 95% confidence level over Rajasthan, Delhi, Punjab, and Himachal Pradesh, whereas CMIP6 MME shows significant rising trends over north India. The rising UTCI trend is also associated with rising surface temperature during summer over the NWI^29^.

The temporal trends in area-averaged ERA5 and CMIP6 MME UTCI are shown in Fig. 3c,d for NWI and the entire Indian region during summer. The temporal variation in UTCI shows an increase in 1998 over NWI and the entire India, which could be due to an extreme heat wave event during 1998^75^. The UTCI increased over NWI after 2000 because the extreme heat wave events mostly happened from 2000 to 2015 in various parts of the country, including NWI^76^. The temporal trend in ERA5 and CMIP6 MME UTCI over NWI and the entire Indian region indicates the rising trends during summer Fig. 3c,d. The CMIP6 MME UTCI trend magnitude (0.21 °C per decade) is higher (~ 62%) than ERA5 for the entire Indian domain (Fig. 3c). The UTCI trend over NWI (0.19 °C per decade (ERA5) and 0.28 °C per decade (CMIP6 MME), Fig. 3d) is higher than entire India (0.08 °C per decade and 0.21 °C per decade). The results indicate that the rising thermal stress is more prominent over NWI compared to the entire Indian region. Results showed that CMIP6 MME reasonably captures thermal stress trend variability like ERA5 over NWI.

**Figure 4:**
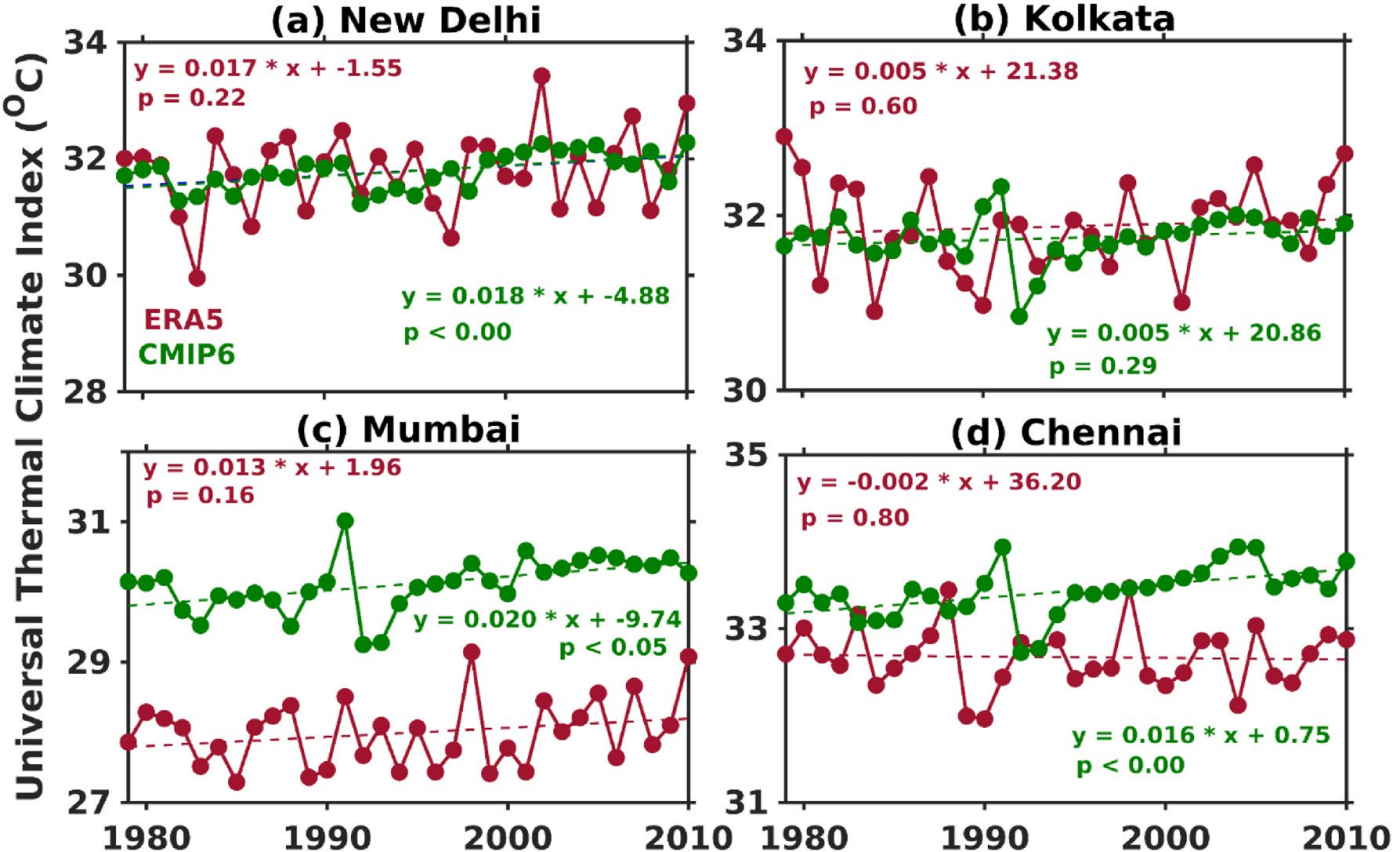
Seasonal temporal trends in ERA5 and CMIP6 MME UTCI at four metropolitan cities (**a**) New Delhi, (**b**) Kolkata, (**c**) Mumbai, and (**d**) Chennai during the summer season. Trends are statistical significance at 95% confidence level.

The urban centres and cities are hotter than the surrounding rural areas due to the urban heat island effect. The cities are becoming warmer day by day due to the factor that release (energy-intensity activities) and trap heat (taller concrete structures) and a lack of natural cooling influence (such as vegetation cover and water sources)^4^. Hence, the investigation of thermal stress is essential at the city scale. These four major metropolitan Indian cities (i.e., New Delhi, Kolkata, Mumbai, and Chennai) have been chosen because the Indian economy and infrastructure mostly depend on these cities^77^. The temporal trends in summertime CMIP6 MME UTCI are also evaluated at the four metropolitan Indian cities (Fig. 4a–d).

**Figure 5:**
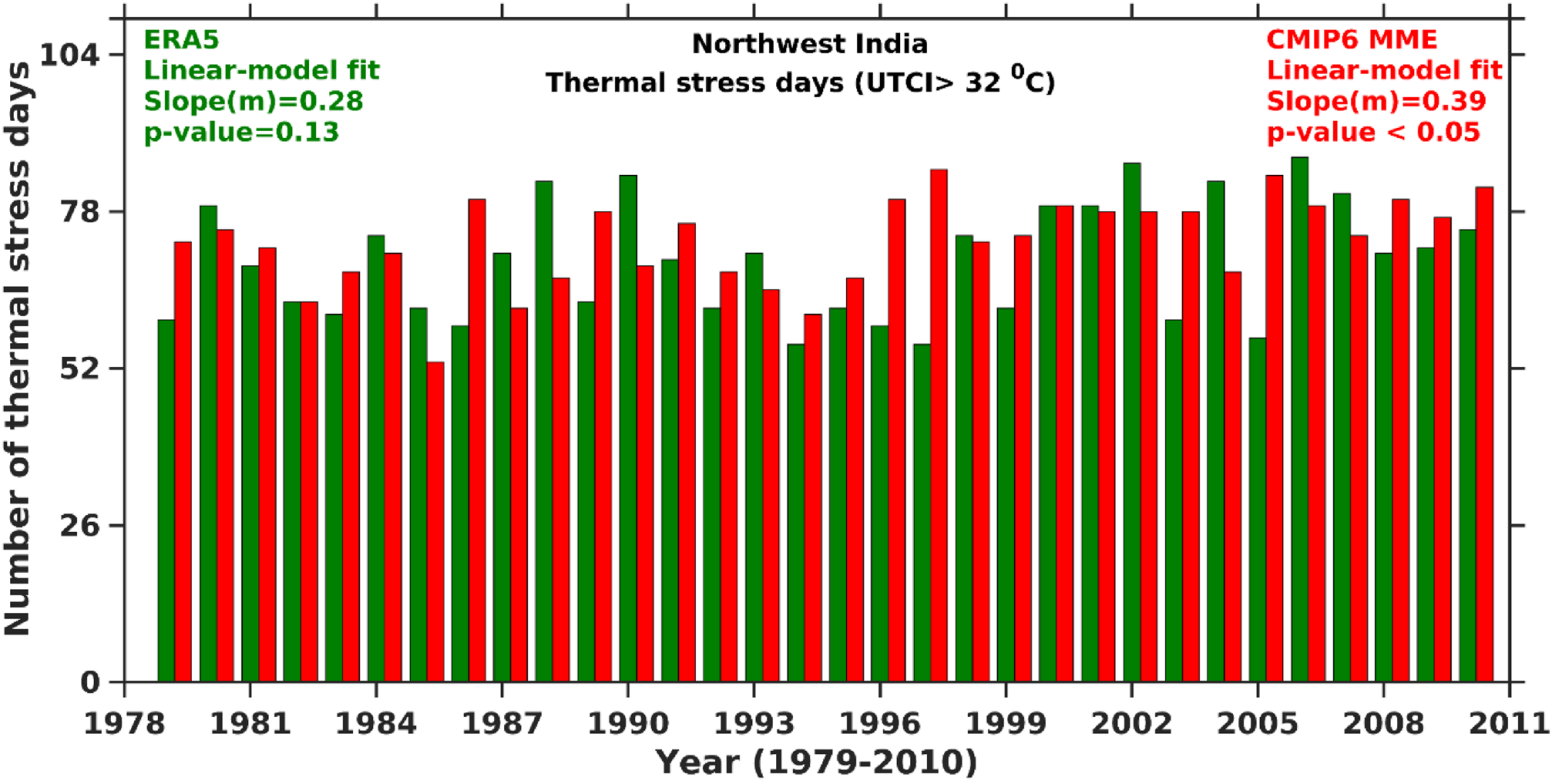
Temporal trends in number of strong thermal stress days from ERA5 and CMIP6 MME UTCI over NWI during summer.

The ERA5 and CMIP6 MME shows increasing UTCI trend over New Delhi, Kolkata, and Mumbai (Fig. 4a–c), while at Chennai ERA5 (decreasing trend) and CMIP6 MME (increasing trend) (Fig. 4d). The magnitude of rising UTCI trends is maximum over New Delhi compared to other cities which demonstrates that the population in New Delhi experiences more thermal discomfort during the hot summer season. Recent study Kumar et al.^78^ shows that Delhi experiences more number of heat wave events during summer than other cities which could be a possible reason for high magnitude of UTCI rising trends. The area-averaged daily UTCI from ERA5 and CMIP6 MME is used to investigate a trend in the strong thermal stress day’s (UTCI > 32 °C) frequency over NWI during different year’s summer (Fig. 5). ERA5 and CMIP6 MME show increasing trends (2.8 (ERA5) and 3.9 (CMIP6 MME) days per decade) in summer thermal stress days over NWI.

### Future changes of human thermal comfort conditions

**Figure 6:**
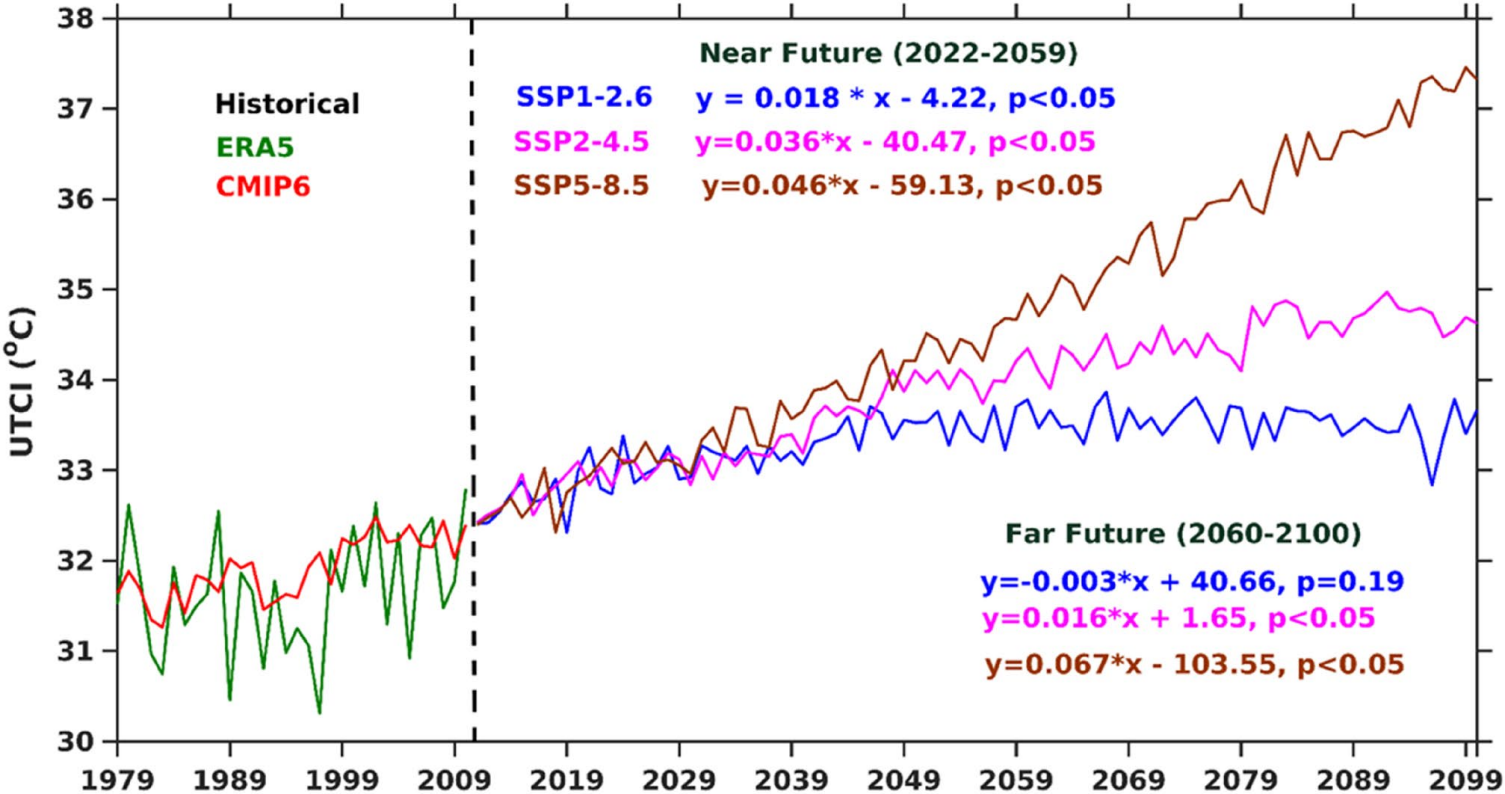
Observed (green) and historical (red) UTCI along with three projected climate scenarios (i.e., SSP1-2.6 (blue), SSP2-4.5 (magenta), and SSP5-8.5 (brown)) over NWI during summer. UTCI trends in all three projected climate scenarios are given for near (2022–2059) and far (2060–2100) future in the Figure.

This section focuses on future projected change in mean UTCI from CMIP6 MME for historical and under three climate scenarios (SSP1-2.6; SSP2-4.5; SSP5-8.5) from different CMIP6 models during the summer season for the period starting from 2011 to 2100 over Indian regional hotspot-NWI. The future climate projections show increase in the summertime annual mean UTCI for three different SSPs. The significant predicted increase in UTCI is noticed 0.2 (SSP1-2.6), 0.3 (SSP2-4.5), and 0.5 (SSP5-8.5) °C per decade over NWI and all these increasing trends are statistically significant at 95% confidence level. The increase of the UTCI is 2.4 °C until the end of this century predicted by SSP1-2.6, and 3.7 °C and 6.1 °C for SSP2-4.5 and SSP5-8.5 (Fig. 6), respectively, during 1979–2100. All three SSPs indicate statistically significant (at 95% confidence level) rising trends (SSP1-2.6 (0.09 °C per decade); SSP2-4.5 (0.26 °C per decade); and SSP5-8.5 (0.56 °C per decade)) in UTCI during summer (2011–2100) over NWI. The historical (1979–2010) ERA5 and CMIP6 MME show increasing trends in UTCI ~ 0.19 (*p*-value = 0.15) and 0.28 (*p* < 0.05) °C per decade, respectively over the hotspot region. Further, the entire period (2011–2100) is divided into three-time spans at intervals of three decades (Table 2). UTCI shows the significant rising trend in near future starting from 2011 to 2040 for SSP1-2.6 (0.23 °C per decade), SSP2-4.5 (0.24 °C per decade), and SSP5-8.5 (0.43 °C per decade); during mid-future (2041–2070) for SSP1-2.6 (0.05 °C per decade), SSP2-4.5 (0.24 °C per decade), and SSP5-8.5 (0.55 °C per decade), and during far-future (2071–2100) for SSP1-2.6 (− 0.05 °C per decade), SSP2-4.5 (0.13 °C per decade), and SSP5-8.5 (0.68 °C per decade).

**Table 2:** Trends in UTCI under three projected climate scenarios.

Trends (°C per decade)	1979–2100		2011–2100		2011–2040		2041–2070		2071–2100	
	Slope	*p*-value	Slope	*p*-value	Slope	*p*-value	Slope	*p*-value	Slope	*p*-value
SSP1-2.6	**0.17**	< 0.05	**0.09**	< 0.05	**0.23**	< 0.05	0.05	0.19	− 0.05	0.24
SSP2-4.5	**0.29**	< 0.05	**0.26**	< 0.05	**0.24**	< 0.05	**0.24**	< 0.05	**0.13**	< 0.05
SSP5-8.5	**0.49**	< 0.05	**0.56**	< 0.05	**0.41**	< 0.05	**0.55**	< 0.05	**0.68**	< 0.05

Significant values are in [bold].

**Figure 7:**
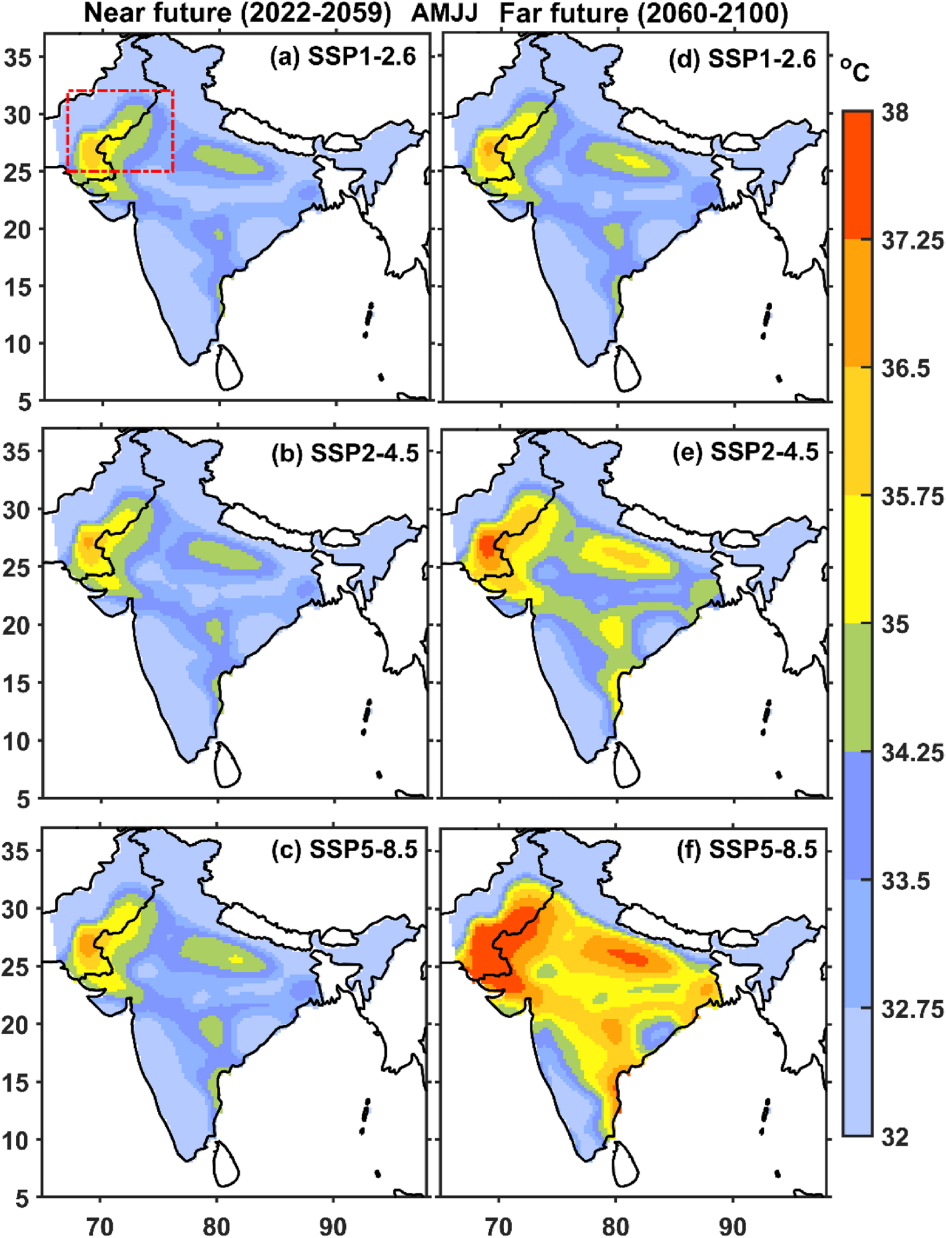
Spatial variation of summertime Multi-Model Ensemble (MME) mean UTCI from all the three projected climate scenarios (i.e., SSP1-2.6, SSP2-4.5, and SSP5-8.5) for the near (2022–2059) and far (2060–2100) future period. The red dotted box over India map highlights the NWI region in (**a**). The map was generated using MATLAB R2017b (www.mathworks.com). The colormap of figures are taken in MATLAB from (https://www.ncl.ucar.edu/Document/Graphics/color_table_gallery.shtml).

Furthermore, the trends in summertime UTCI are also investigated for the near future (2022–2059) and far future (2060–2100) under all three projected climate scenarios over NWI, which is given in Fig. 6. All the three projected climate scenarios for the near future showed significant rising trends in UTCI over NWI, which are 0.18 °C per decade (SSP1-2.6), 0.36 °C per decade (SSP2-4.5), and 0.46 °C per decade (SSP5-8.5). The projected rising trends magnitude in UTCI under SSP2-4.5 and SSP5-8.5 scenarios is ~ two and threefold of the SSP1-2.6 scenario, respectively. The projected UTCI increasing trends are only significant for two climate scenarios, SSP2-4.5 (0.16 °C per decade) and SSP5-8.5 (0.67 °C per decade) for the far future period. The projected rising trends magnitude in UTCI under the SSP5-8.5 scenario for the far future is higher than the near future, and under the SSP2-4.5 scenario for the far future is less than the near future. The summer thermal stress projection has been investigated in the near (Fig. 7a–c) and the far future (Fig. 7d–f) over India. All the MME mean UTCI under the projected climate scenarios show strong thermal stress (> 35 °C) conditions in the near future over NWI, whereas moderate thermal stress conditions (~ 32 °C) over the rest of India. The thermal stress intensity over NWI gradually increases under these two projected scenarios (SSP1-2.6 and SSP2-4.5), whereas becomes maximum in the SSP5-8.5 scenario in the near future (Fig. 7a–c). The SSP1-2.6 scenario shows the strong thermal stress over NWI and across IGP region. Similarly, the thermal stress gradually increases from strong thermal stress (SSP2-4.5 scenario, Fig. 7e) to very strong thermal stress (SSP5-8.5 scenario, Fig. 7f) in the far future.There would be a significant impact over NWI with the presence of extremely high thermal stress under the SSP5-8.5 scenario. Additionally, the IGP and central Indian region will also likely experience strong thermal stress during the hot season.

**Figure 8:**
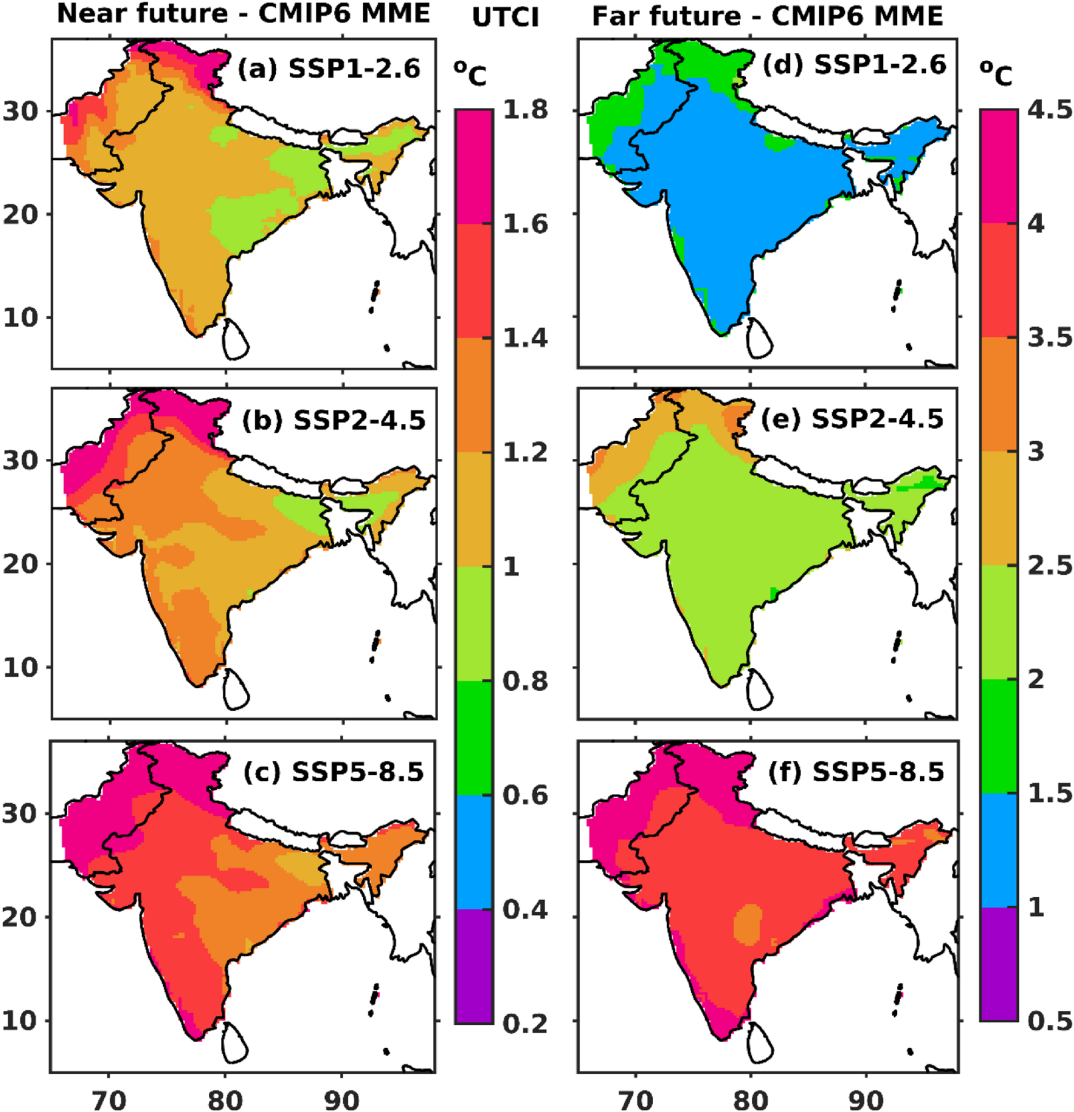
Multi-Model Ensemble (MME) mean projected changes in summertime UTCI in all the three climate scenarios for the near (2022–2059) and far (2060–2100) future against the historical reference period (1979–2010). The map was generated using MATLAB R2017b (www.mathworks.com). The colormap of figures are taken in MATLAB from (https://www.ncl.ucar.edu/Document/Graphics/color_table_gallery.shtml).

MME mean projected changes in summertime UTCI from CMIP6-GCMs in all three climate scenarios for the near and far future against the historical reference period (1979–2010) is presented in Fig. 8. All three projected climate scenarios for the near and far future is generally higher than the historical period. The changes between all the projected scenarios and historical vary less than 2 °C (for the near future, Fig. 8a-c) and between 3 and 5 °C (for the far future, Fig. 8d–f). Various researchers^79,80^ have previously reported a notable increase in the evaporation of moisture and water vapour contents from the ocean, attributed to a significant rise in the sea surface temperature. Consequently, the movement of moisture-rich winds along with surface temperature rise, which gives way to hot and humid conditions that impose thermal stress on the NWI region^29^.

**Figure 9:**
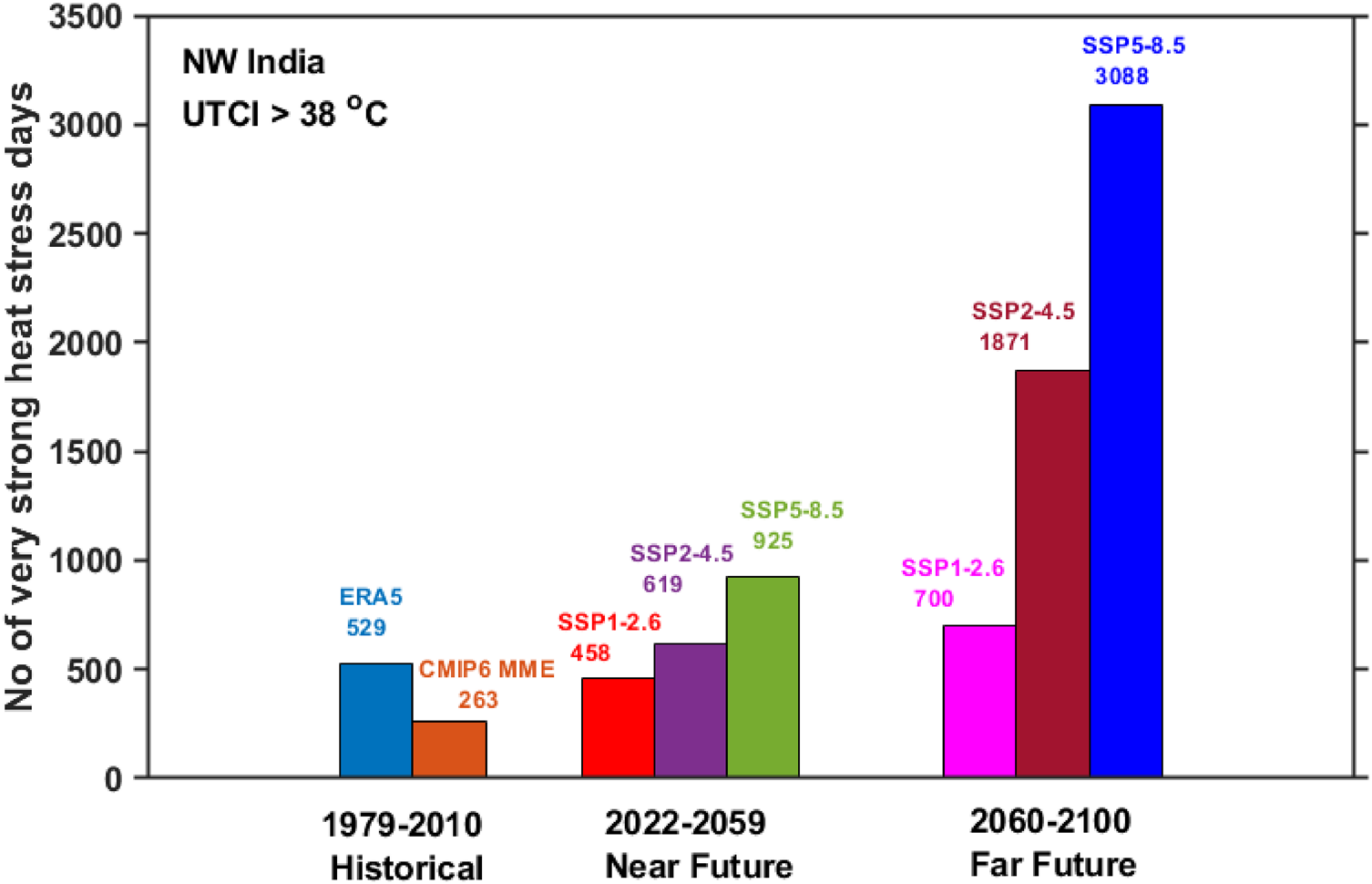
Summertime very strong heat stress days (UTCI > 38 °C) over NWI during historical and for all the three projected climate scenarios in near and far future.

Moreover, the occurrence of very strong thermal stress days (UTCI > 38 °C) is examined using summertime ERA5 and CMIP6 MME UTCI over NWI during the historical period, as well as under various projected climate scenarios in the near and far future (Fig. 9). The ERA5 and CMIP6 MME shows 529 and 263 very strong thermal stress days over the hotspot region, respectively. CMIP6 MME has been projected to increase strong thermal stress days significantly over the twenty-first century. The number of very strong thermal stress days gradually increases under SSP1-2.6 (458 days), SSP2-4.5 (619 days), and SSP5-8.5 (925 days) scenarios in near future. There is a two- or three-fold increase in very strong thermal stress days from near future to far future (SSP1-2.6 (700 days), SSP2-4.5 (1871 days), and SSP5-8.5 (3088 days)) under all the project climate scenarios. The number of very strong thermal stress days in the far future rises three-fold of the days in the near future under the SSP2-4.5 and SSP5-8.5 scenarios. The number of very strong thermal stress days under the SSP5-8.5 scenario in the near future is about two times the historical days. An unprecedented (about 6 times the historical days) increase in very strong thermal stress days is noticed in the far future. The study's finding suggests that NWI will likely witness an unprecedented rise in the very strong thermal stress days. Saeed et al.^81^ concluded, based on wet-bulb temperature, that South Asia, especially IGP, experiences deadly thermal stress at 1.5 °C of global warming. Kumar et al.^78^ reported strong to very strong thermal stress over Delhi, a highly populated metro city in NWI, during summer (1990–2019). Shukla et al.^29^ showed significant rising trends in UTCI (1981–2019) over NWI and strong thermal stress over the study region. Kumar and Sharma^39^ demonstrate very strong heat stress conditions over a semi-arid site in NWI. Various studies have concluded the strong to very strong thermal stress conditions based on UTCI over NWI, which also aligns with the present study's findings.

The increasing occurrence of highly intense thermal stress days will pose significant risks to both human health and crop production in the region. Moreover, it will have adverse effects on the terrestrial ecosystem, contributing to forest fires and droughts^82^. The summer/hot season in tropical and sub-tropical countries brings about environmental heat conditions that have detrimental effects on multiple sectors, including human health, agriculture, the ecosystem, and the economy. Indoor thermal stress is primarily caused by local heat sources, such as glass and metal factories^83,84^. Outdoor workers, including those in agriculture, mining, quarrying, construction, and shipbuilding, face thermal stress resulting from intense sunlight and hot environmental conditions during summer^39,85–88^. Thermal stress can lead to physiological issues due to elevated core body temperature, ranging from mild symptoms like skin rash, sweating, dehydration, and salt loss to more severe consequences such as heat exhaustion, reduced productivity, heat stroke, unconsciousness, and even mortality^32,33,89,90^. Furthermore, thermal stress has a negative impact on agriculture and the ecosystem, significantly affecting crop production^91–93^ (. It restricts crop growth and metabolism, resulting in significant yield losses. Rao et al.^44^ reported a decline of 30 to 40% in work performance in India due to thermal stress, which has detrimental effects on the country's economy. Matsumoto et al.^94^ demonstrated a decline in labor productivity in India due to increasing levels of thermal stress, further impacting the economy. Therefore, the development of appropriate guidelines to address these issues is crucial for improving the health, productivity, and overall economy of indoor and outdoor workers. This study underscores the importance of implementing thermal stress management plans to mitigate adverse impacts across various sectors.

## Conclusions

The present study evaluates the capability of CMIP6 to assess thermal stress condition over NWI during summer. The findings of the present study are summarized as follows.

- The spatial pattern of the summertime climatological (1979–2010) mean of ERA5 UTCI shows the strong thermal stress over a hotspot region (i.e., NWI). The CMIP6 MME reasonably captures the strong thermal stress like ERA5 over NWI. The spatial trends in UTCI (ERA5 and CMIP6 MME) show the rising trends over NWI. The CMIP6 MME shows more significant trends than ERA5 and trend magnitude is usually greater than 0.2 °C per decade.
- The area-averaged summertime ERA5 and CMIP6 MME UTCI for the entire India and NWI depict that the UTCI rising trend magnitude is higher over NWI than entire India. It demonstrates that NWI experiences more strong thermal stress than India. The summertime UTCI trends over New Delhi are higher than in other metropolitan cities (i.e., Kolkata, Mumbai, and Chennai), which could be due to frequent and more number of summertime heat wave events over the region.
- The number of summertime thermal stress days (UTCI > 32 °C) from ERA5 and CMIP6 MME shows the increasing trends over NWI. The rising trends in the thermal stress days are ~ 2.8 days per decade (ERA5) and 3.9 days per decade (CMIP6 MME) over the study regions.
- The future climate projections show an increase in the mean UTCI under all three projected climate scenarios over NWI during summertime. The results showed a significant rising trend in summertime CMIP6 MME UTCI under different projected scenarios (2011 to 2100) for SSP1-2.6 (0.9 °C per decade), SSP2-4.5 (0.26 °C per decade), and SSP5-8.5 (0.56 °C per decade), respectively.
- All three projected scenarios show the strong thermal stress (> 35 °C) condition in the near future over NWI, whereas moderate thermal stress condition (~ 32 °C) over rest part of India. Similarly, the thermal stress gradually increases from strong thermal stress (SSP2-4.5 scenario) to very strong thermals stress (SSP5-8.5 scenario) in the far future. SSP5-8.5 scenario in far future affects the NWI with very strong thermal stress and in the IGP and central Indian region with strong thermal stress.The changes in the UTCI from all three projected scenarios in the near and far future are always higher than in the historical period, and the differences vary between 1 to 5 °C. The number of summertime very strong heat stress days (UTCI > 38 °C) over NWI in the near future showed a two to three-fold rise in the far future under all the projected future scenarios.

Overall, the present study reveals a notable increase in the frequency of intense thermal stress days over NWI during the summer season. Consequently, it highlights the urgent need to evaluate the future implications of such severe heat stress conditions on human health in the NWI region. To mitigate the adverse effects on human well-being, the implementation of an early warning system based on thermal stress assessment becomes crucial. Therefore, it is essential to further develop and deploy AI/ML-based models that can accurately predict local-scale thermal stress conditions.

The limitation of the present study is the coarse resolution of all CMIP6 models because it can mask important local features and phenomena, such as extreme heat stress events, orographic effects, or land-sea contrasts. Moreover, climate model (CMIP6) results and interpretation, particularly in future scenarios, have inherent limitations in terms of reliability. Assessing the accuracy of data for different models by comparing them to observations in historical results provides some insight, but it is unclear whether this fully validates their accuracy in a changing future climate. Some studies have highlighted and discussed limitations in the interpretation of heat stress indicators such as UTCI^17,47^.

## Data Availability

Data used in the present study is available in public domain and can be downloaded from the website link: https://cds.climate.copernicus.eu/cdsapp#!/dataset/sis-extreme-indices-cmip6?tab=overview
